# A Prospective Study of Negative Pressure Wound Therapy With Integrated Irrigation for the Treatment of Diabetic Foot Ulcers

**Published:** 2011-02-16

**Authors:** Charles M. Zelen, Brian Stover, David Nielson, Muriel Cunningham

**Affiliations:** ^a^Professional Education and Research Institute, Roanoke, VA; ^b^Department of Orthopedics, HCA Lewis-Gale Medical Center, Salem, VA

## Abstract

**Objective:** Patients with diabetes often present with pedal wounds resistant to standard wound healing modalities and become chronic in nature. These chronic wounds in diabetic patients have a high incidence of complications including infection and amputation. Negative pressure wound therapy has been found to facilitate healing of the stagnant pedal wound. This protocol was designed to determine wound closure rates using a unique negative pressure wound therapy system that delivers vacuum-assisted wound closure with a simultaneous irrigation feature (Svedman Wound Treatment System). **Methods:** A prospective single center study was conducted in adults with diabetic foot ulcers ≥cm^2^ or more in size showing no signs of clinical infection, and having adequate blood flow. Patients received dressing changes and irrigation on a standard regimen with weekly wound assessments for a minimum of 6 weeks. **Results:** 11 women and 8 men with a mean wound size of 2.4 cm × 2.2 cm were treated with the device. A total of 14 of /19 (74%) patients healed completely, with a median healing time of 34 days (range, 9-114). Eleven of 19 patients (58%) healed within the 6-week evaluation period. For the 5 patients who did not heal completely with the device, other treatments were utilized, including further wound debridement, muscle flaps, and skin grafting procedures. **Conclusions:** Negative pressure wound therapy with integrated irrigation was well tolerated by the patients without complications related to the device application or irrigation feature. The data clearly suggests that this technology may be a promising alternative for the chronic nonhealing diabetic wound.

The rising prevalence of diabetes is a well-established fact; the number of diagnosed patients is tripling in the United States from 1980 to 2007.^1^ As of 2007, 23.6 million people in the United States had diabetes, which is 7.8% of the total population.^2^ The consequences of foot ulcers associated with diabetic neuropathy include morbidity and mortality as well as significant increases in the health care costs of this patient population. Of those 40 years and older with diabetes, 30% experience reduced sensation in their feet,^2^ and the annual cost of diabetic neuropathy is estimated to be $10.91 billion in the United States alone.^3^ In patients with diabetes, the age-adjusted prevalence of a history of foot ulcers is 12.7% (95% CI: 11.9-13.6),^4^ some of these wounds resulting in complications such as the need for lower extremity amputations. In the United States, approximately 71000 nontraumatic amputations of the lower limb occurred in patients with diabetes in 2004.^2^

Previous studies in patients with diabetes have demonstrated that negative pressure wound therapy (NPWT) facilitates healing in difficult wounds and reduces healing times^5^^-^7 resulting in lower health care costs.^7^,^8^ The purpose of this trial was to determine the efficacy of NPWT with irrigation in the diabetic wound utilizing the Svedman Wound Treatment System (Svedman Wound Treatment System, Innovative Therapies, Inc., Gaithersburg, Maryland). This serves as the first prospective clinical study using the Svedman device. The prospective single center single arm trial was instituted using the Svedman Wound Treatment System with irrigation^9^ in a series of 20 diabetic patients with chronic nonhealing foot ulcers. Wound closure rates and outcomes were assessed, in addition to the safety of the device.

## METHODS

This was a 6-week, single arm, prospective trial. The study protocol was approved by the Western Institutional Review Board and informed consent was obtained from all patients prior to enrollment. The primary objective of the study was to determine the wound closure rates using the Svedman Wound Treatment System with irrigation over the 6-week evaluation period. Secondary objectives included assessment of the proportion of healing at 6 weeks and the safety of the device. Foam dressings were changed 3 times weekly with irrigation at the time of dressing change. A total of 20 patients were consented for the trial with 19 qualifying for active treatment. The patient that was unable to participate in active treatment was found to have an ulceration less than 1 cm^2^ after debridement and therefore excluded. To obtain a representative sample, diabetic wound patients presenting to the investigator who had wounds that failed to respond to conservative treatment for at least 4 weeks were offered the opportunity to join the study if they met inclusion and exclusion criteria. Eligible patients were men and women older than >18 years diagnosed with type 1 or type 2 diabetes mellitus and neuropathy with ulceration. The patients' foot ulcer was to be ≥ cm^2^ or more in size at any location on the foot with no clinical signs of infection that had failed a minimum of 4 weeks of conservative care. Wounds were measured postdebridement to determine the wound size inclusion criterion. Any patients with ulcers probing to bone (UT Grade IIIA-B)^10^ were excluded. Patients were also excluded if they were undergoing dialysis treatment for kidney failure, had a history of poor compliance with medical treatments, were participating in another clinical trial, were receiving radiation therapy or chemotherapy, had a known or suspected malignancy near the index ulcer, had an ankle-brachial index less than 0.7, or were without a pulse detectable by Doppler.

At the initial visit (day 0), wounds were debrided and a baseline measurement was obtained, including the percentage of necrotic tissue (linear scale, 0%-100%). The wound was measured with a plastic metric ruler and a digital photograph of the wound was taken at a distance of 30 cm. Each wound also underwent a 3-dimensional imaging and acetate tracing study. The NPWT system was then applied in a manner consistent with the manufacturer's instructions (Svedman Wound Treatment System, Innovative Therapies, Inc., Gaithersburg, Maryland).^11^ Briefly, the wounds were debrided as medically necessary and a piece of ITI Svedman porous wound foam was cut to the size of the pedal wound. The inflow tubing/lavage catheter and the outflow tubing/suction catheter were then covered with an adhesive dressing, Usage of additional foam for placement of the tubing/catheters may be needed. Suction cup applicators have since been added to the dressing system but for the purpose of this trial the institutional review board recognized and approved the original dressing with the tubing/catheter directly inserted into the foam. The suction tubing was clamped and used for lavage of saline before dressing changes. Continuous pressure was applied using the default setting of 120 mm Hg. Each patient was provided with a device user's manual and was instructed on to how to operate the apparatus and care for their wound. Patients were offloaded using a removable cast walker (cbiTCC, Ossur Medical, Inc, Camarillo, California).

**Figure 1:**
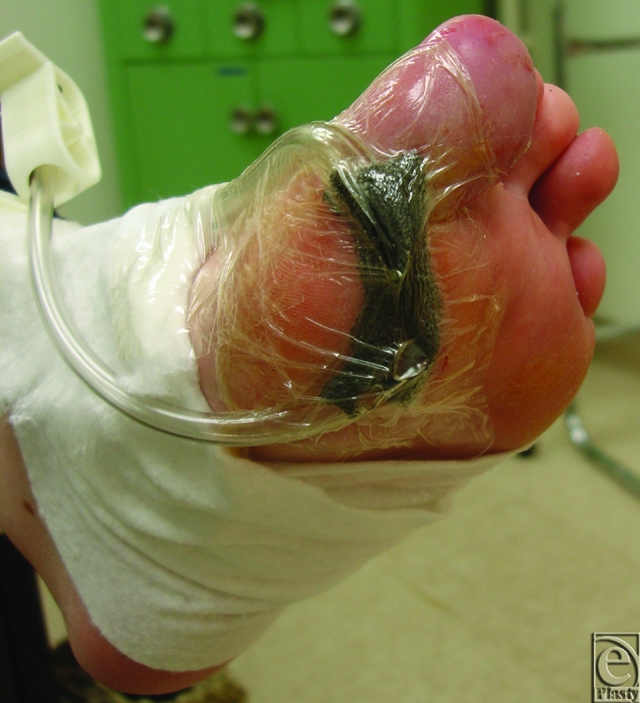
The Svedman Wound Treatment System foam dressing and catheters on a plantar first metatarsal pedal wound.

To maintain the very best reproducibility, all dressing changes were performed at the research site by the investigator, subinvestigator, or research nurse. Dressings were changed every second or third day with the majority of all patients returning for dressing changes every Monday, Wednesday, and Friday. The progress of wound healing was measured weekly with acetate 3-dimentional tracings. Before removing dressings, wounds were irrigated simultaneously with 1000 cc sterile normal saline via the Svedman Wound Treatment System and the irrigation tubing (Irrigation Tubing with SpeedConnect, Innovative Therapies, Inc.). Upon removal of the dressing, sharp debridement was performed as needed and the wounds were measured and photographed as previously described. These measurements were collected until the end of the study, wound healing, or patient withdrawal. All dressing changes performed in between the regular weekly visits were recorded by study personnel and documented in the source documentation. Patients had the option of continuing NPWT if they had not completely healed by the end of 6-week evaluation period. All patients who were benefiting from treatment chose to continue therapy. A clinical photograph of the Svedman Wound Treatment dressing and device is presented in Figure 1.

## RESULTS

A total of 20 patients were enrolled at a single diabetic foot clinic in the United States. Enrollment was concluded after 20 patients. Treatment was not initiated for 1 white 57-year-old male patient because of a small wound size at the baseline visit. He was consented for participation in the study, but after debridement, the wound margins were more epithelialized than initially expected, and the resultant wound size did not meet inclusion criteria. The patient was therefore excluded and never received active treatment.

Patient characteristics, study disposition, and wound healing over time are presented in Table 1. A total of 11 women and 8 men were enrolled, 89% white (*n* = 17) and 11% African American (*n* = 2). The median patient age was 64 years, ranging from 43 to 81. Approximately, half of the wounds occurred on the right lower limb and half on the left lower limb. The most frequently reported wound locations were right plantar in 4 of /19 (21%) cases, followed by the right heel and left heel with 3 of /19 (16%) cases each. Wound sizes at the initial visit ranged from 1.0 cm × 1.0 cm to 5.0 cm × 7.0 cm, with a mean wound size of 2.4 cm × 2.2 cm. The median baseline necrosis was 50% (range, 15-100).

**Figure 2:**
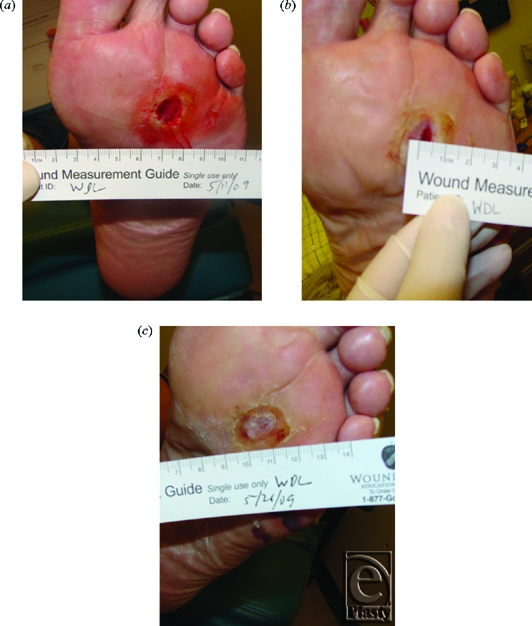
A representative patient with a plantar forefoot wound that healed within 3 weeks. (*a*) Wound at study entry; (*b*) After 1 week of negative pressure therapy with irrigation; (*c*) Complete healing after second week of therapy.

A total of 14 of /19 (74%) patients healed completely using NPWT, with a median time to healing of 34 days (range, 9-124). Eleven of 19 patients (58%) healed within the 6-week evaluation period. Of those patients healing within 6 weeks, the median baseline wound size was 2.52 cm^2^ (range, 1.0-13.72 cm^2^) and the median baseline amount of necrosis was 40% (range, 15%-80%). Photographs of a representative patient with a plantar forefoot wound that healed within 3 weeks are presented in Figures 2a to 2c.

Three of the 5 remaining patients required additional interventions. Patient 5 required a skin graft on day 88 to complete healing and was healed on day 113. Patient 6 underwent a muscle flap and skin graft on day 61, completely healing on day 124. Patient 17 had the largest wound (5.0 cm × 7.0 cm) in the study with 100% necrosis at baseline. This patient underwent 2 split-thickness wound grafts after initial NPWT therapy with 60% successful graft take to the wound but still had not healed as of day 135 and was referred to an outside specialist for treatment.

One serious adverse event was reported with patient 12 who experienced cellulites and infection after 12 days in the study. She was admitted to the hospital and although she was given ample options for limb salvage, having a long history of diabetic ulcers and infections with her foot, the patient opted for limb amputation. The serious adverse event was unrelated to the negative pressure therapy device.

The study procedures and NPWT device were well tolerated by the study patients. Both the NPWT system and the irrigation feature functioned well in all patients enrolled without any known adverse events related to the unit.

## DISCUSSION

In this prospective study conducted in a clinic setting, NPWT using the Svedman Wound Treatment System with irrigation was successful in facilitating wound closure and healing in both large and small wounds resulting from complications of diabetic neuropathy and pressure on the distal lower limb. In the smaller wounds, NPWT led to rapid epithelialization with 1 patient experiencing complete healing as quickly as 9 days. In more complicated cases with larger wounds, complete healing occurred with NPWT, and in some cases, NPWT with the Svedman system provided a catalyst to create a beneficial granular bed for tissue grafting in an area that had very little viable tissue at the beginning of therapy. Therefore, the Svedman Wound Treatment System may not only be a viable option for both granulation and epithelialization of a diabetic wound but may also serve as a catalyst to grow granular tissue and facilitate other procedures that may lead to complete wound healing.

Preventing diabetic ulcers from progressing in depth and becoming infected and developing into more serious conditions is not only clinically beneficial but has a positive impact on health care resources. In a retrospective study of claims data from patients with diabetes, Stockl et al^12^ reported that patients who experienced complications such as wounds probing to bone or those requiring amputation had greater costs related to their ulcers when compared with patients who did not progress (US$20 136 vs US$3063, respectively; *P* < .0001). In a recent prospective study conducted in Europe, patients whose foot ulcers healed had an associated cost of €7722 vs €20064 for patients who did not heal within a 12-month time frame.^13^ Given the high costs of nonhealing wounds and the associated negative impact on patient quality of life, outpatient interventions that prevent ulcer progression and promote healing are of benefit to patients and clinicians.

This study enrolled a small sample with a large number of the wounds being smaller than the mean wound size and did not have a comparator group; yet, several useful conclusions may be derived. The procedures and device were well tolerated by the study patients, who made up a diverse and representative convenience sample treated in a real-world setting. In addition, patients who healed during the study had diverse wound sizes and locations, suggesting that the device may be utilized in a variety of locations in the diabetic foot. Overall, the study results suggest that the Svedman Wound Treatment System was effective in wounds of all sizes, both large and small, in not only providing outstanding granulation but also significant epithelialization with the majority of wounds healing completely without any other modality applied. Further studies involving the irrigation feature should be considered, with daily irrigation possibly providing additional benefit not only to the diabetic wound but also posttraumatic wounds and decubitus ulceration. In addition, a study with a larger mean wound size may be considered, although most pedal wounds are by nature small but difficult to heal with standard therapy. Patient compliance also plays a large role in wound healing. Although the patients were each given an offloading boot with strict instructions to remain in the boot with any ambulation, future studies may consider an activity-monitoring device to better assess patient compliance. Lastly, a comparator trial to other forms of negative pressure wound management may be considered to determine whether the Svedman Wound Treatment System with its irrigation feature could demonstrate superiority.

## Acknowledgments

The authors thank Krissy Mather-Mullins, RN and clinical research nurse, for her assistance with the clinical trial and Brandy Nicole Scott, BSN and clinical research nurse for her assistance with final data preparation and manuscript revision. Financial support for the study and manuscript was provided by Innovative Therapies, Inc. Gaithersburg, MD.

## Figures and Tables

**Table 1:** Patient characteristics, study disposition, and wound healing over time: all treated patients*

Patient Number	Age, y	Gender	Baseline Wound Size, cm	Wound Location	Necrotic Baseline, %	Necrotic Day 14, %	Necrotic Day 28, %	Time to Healing (Days) or Other Outcome
				Patients completing the study (*n* = 15)				
13	43	F	1.2 × 0.9	Rt hallux	15	–	–	9
20	61	F	1.5 × 1.5	Rt plantar	40	–	–	9
9	60	F	1.2 × 2.0	Rt plantar	20	–	–	12
18	67	M	1.4 × 1.8	Lt forefoot	15	0†	–	15
2	45	F	1.2 × 1.5	Lt hallux	80	5	–	19
4	81	M	2.1 × 2.2	Lt hallux	70	40	–	26
8	53	F	1.6 × 2.2	Lt midfoot	40	40	20	32
1	65	F	4.9 × 2.8	Rt heel	60	20	9	35
11	64	M	1.0 × 1.0	Lt plantar	50	25	0	35
16	64	F	4.5 × 1.0	Lt heel	25	10	10	35
3	71	M	2.1 × 1.4	Rt heel	30	5	0	40
7	72	M	2.6 × 3.1	Lt heel	98	30	15	65
5	49	M	2.7 × 4.1	Lt midfoot	50	25	20	113
6	57	F	1.7 × 1.0	Rt midfoot	90	70	20	124
17	64	M	5.0 × 7.0	Lt heel	100	95	80‡	Did not heal after 135 days
				Patients discontinuing from the study (*n* = 4)				
12	65	F	2.0 × 1.3	Rt plantar	90	–	–	Withdrew after 12 days due to an SAE of cellulites
14	72	F	1.0 × 1.0	Rt plantar	50	90	–	Withdrew after 21 days due to lack of progress
15	74	M	2.4 × 2.0	Lt dorsal	95	50	25	Withdrew after 40 days to pursue other treatment options
19	64	F	5.5 × 3.7	Rt heel	100	85	95	Withdrew after 40 days of NPWT

*– indicates healed or withdrew prior to this visit; F, female; ID, identification; lt, left; M, male; NPWT, negative pressure wound therapy; rt, right; SAE, serious adverse event.

†Measured on day 15.

‡Measured on day 29.
